# Supplementary material for: Relationships among creativity indices: Creative potential, production, achievement, and beliefs about own creative personality
Source: PLoS One. 2022 Sep 28;17(9):e0273303. doi: 10.1371/journal.pone.0273303 (PMC9518913; doi:10.1371/journal.pone.0273303)
Supplement: S1 Table — (DOCX) [file pone.0273303.s001.docx]

**S1 Table. Results of Multiple Regression Analysis in the effect of flexibility with Creative Production Score as the Objective Variable.**

| **Predictors** |  | **95% CI** | |  |  |  |  |
| --- | --- | --- | --- | --- | --- | --- | --- |
|  | ***b*** | **LL** | **UL** | ***T*** | **df** | ***p*** | ***β*** |
| Step 1 (*R*^2^ = .103, *p* = .003) |  |  |  |  |  |  |  |
| Intercept | 3.27 | 3.16 | 3.38 | 58.9 | 84 | < .0001 |  |
| S-A Creativity test (flexibility) | 0.03 | 0.013 | 0.057 | 3.1 | 84 | .003 | .32 |
| Step 2 (*R*^2^ = .120, *p* = .005) |  |  |  |  |  |  |  |
| Intercept | 3.27 | 3.16 | 3.38 | 59.1 | 83 | < .0001 |  |
| S-A Creativity test (flexibility) | 0.03 | 0.009 | 0.055 | 2.8 | 83 | .006 | .30 |
| CPS | 0.17 | -0.097 | 0.44 | 1.3 | 83 | .209 | .13 |
| Step 3 (*R*^2^ = .153, *p* = .003) |  |  |  |  |  |  |  |
| Intercept | 3.29 | 3.18 | 3.40 | 59.2 | 82 | < .0001 |  |
| S-A Creativity test (flexibility) | 0.03 | 0.01 | 0.055 | 2.9 | 82 | .005 | .30 |
| CPS | 0.20 | -0.07 | 0.46 | 1.5 | 82 | .147 | .15 |
| S-A Creativity test (flexibility)  × CPS | -0.05 | -0.096 | 0.005 | -1.8 | 82 | .077 | -.18 |

CI: confidential interval, LL: lower limits, UL: upper limits, CPS: creativity personality scale
