# Supplementary material for: Relationships among creativity indices: Creative potential, production, achievement, and beliefs about own creative personality
Source: PLoS One. 2022 Sep 28;17(9):e0273303. doi: 10.1371/journal.pone.0273303 (PMC9518913; doi:10.1371/journal.pone.0273303)
Supplement: S2 Table — (DOCX) [file pone.0273303.s002.docx]

**S2 Table. Results of Multiple Regression Analysis in the effect of originality with Creative Production Score as the Objective Variable.**

| **Predictors** |  | **95% CI** | |  |  |  |  |
| --- | --- | --- | --- | --- | --- | --- | --- |
|  | ***b*** | **LL** | **UL** | ***T*** | **df** | ***p*** | ***β*** |
| Step 1 (*R*^2^ = .065, *p* = .018) |  |  |  |  |  |  |  |
| Intercept | 3.27 | 3.16 | 3.38 | 57.7 | 84 | < .0001 |  |
| S-A Creativity test (originality) | 0.03 | 0.006 | 0.060 | 2.4 | 84 | .018 | .25 |
| Step 2 (*R*^2^ = .090, *p* = .020) |  |  |  |  |  |  |  |
| Intercept | 3.27 | 3.16 | 3.38 | 58.1 | 83 | < .0001 |  |
| S-A Creativity test (originality) | 0.03 | 0.003 | 0.057 | 2.2 | 83 | .029 | .23 |
| CPS | 0.21 | -0.064 | 0.47 | 1.5 | 83 | .133 | .16 |
| Step 3 (*R*^2^ = .099, *p* = .036) |  |  |  |  |  |  |  |
| Intercept | 3.28 | 3.16 | 3.39 | 57.8 | 82 | < .0001 |  |
| S-A Creativity test (originality) | 0.03 | 0.003 | 0.057 | 2.2 | 82 | .032 | .23 |
| CPS | 0.22 | -0.053 | 0.488 | 1.6 | 82 | .114 | .17 |
| S-A Creativity test (originality) × CPS | -0.03 | -0.083 | 0.031 | -0.9 | 82 | .373 | -.09 |

CI: confidential interval, LL: lower limits, UL: upper limits, CPS: creativity personality scale
