# Supplementary material for: Relationships among creativity indices: Creative potential, production, achievement, and beliefs about own creative personality
Source: PLoS One. 2022 Sep 28;17(9):e0273303. doi: 10.1371/journal.pone.0273303 (PMC9518913; doi:10.1371/journal.pone.0273303)
Supplement: S3 Table — (DOCX) [file pone.0273303.s003.docx]

**S3 Table. Results of Multiple Regression Analysis in the effect of flexibility with Creative Achievement Score as the Objective Variable.**

| **Predictors** |  | **95% CI** | |  |  |  |  |
| --- | --- | --- | --- | --- | --- | --- | --- |
|  | ***b*** | **LL** | **UL** | ***T*** | **df** | ***p*** | ***β*** |
| Step 1 (*R*^2^ = .054, *p* = .030) |  |  |  |  |  |  |  |
| Intercept | 1.66 | 1.47 | 1.84 | 17.8 | 85 | < .0001 |  |
| S-A Creativity test (flexibility) | 0.04 | 0.004 | 0.078 | 2.2 | 85 | .030 | .23 |
| Step 2 (*R*^2^ = .091, *p* = .018) |  |  |  |  |  |  |  |
| Intercept | 1.66 | 1.48 | 1.84 | 18.1 | 84 | < .0001 |  |
| S-A Creativity test (flexibility) | 0.03 | -0.002 | 0.072 | 1.9 | 84 | .066 | .20 |
| CPS | 0.41 | -0.03 | 0.85 | 1.8 | 84 | .069 | .19 |
| Step 3 (*R*^2^ = .156, *p* = .003) |  |  |  |  |  |  |  |
| Intercept | 1.62 | 1.44 | 1.80 | 18.0 | 83 | < .0001 |  |
| S-A Creativity test (flexibility) | 0.03 | -0.003 | 0.070 | 1.8 | 83 | .068 | .19 |
| CPS | 0.36 | -0.073 | 0.791 | 1.7 | 83 | .102 | .17 |
| S-A Creativity test (flexibility)  × CPS | 0.10 | 0.022 | 0.187 | 2.5 | 83 | .013 | .26 |

CI: confidential interval, LL: lower limits, UL: upper limits, CPS: creativity personality scale
