# Supplementary material for: Relationships among creativity indices: Creative potential, production, achievement, and beliefs about own creative personality
Source: PLoS One. 2022 Sep 28;17(9):e0273303. doi: 10.1371/journal.pone.0273303 (PMC9518913; doi:10.1371/journal.pone.0273303)
Supplement: S4 Table — (DOCX) [file pone.0273303.s004.docx]

**S4 Table. Results of Multiple Regression Analysis in the effect of originality with Creative Achievement Score as the Objective Variable.**

| **Predictors** |  | **95% CI** | |  |  |  |  |
| --- | --- | --- | --- | --- | --- | --- | --- |
|  | ***b*** | **LL** | **UL** | ***T*** | **df** | ***p*** | ***β*** |
| Step 1 (*R*^2^ = .030, *p* = .107) |  |  |  |  |  |  |  |
| Intercept | 1.66 | 1.47 | 1.85 | 17.6 | 85 | < .0001 |  |
| S-A Creativity test (originality) | 0.04 | -0.008 | 0.082 | 1.6 | 85 | .107 | .17 |
| Step 2 (*R*^2^ = .075, *p* = .038) |  |  |  |  |  |  |  |
| Intercept | 1.66 | 1.48 | 1.84 | 18.0 | 84 | < .0001 |  |
| S-A Creativity test (originality) | 0.03 | -0.013 | 0.076 | 1.4 | 84 | .163 | .15 |
| CPS | 0.45 | 0.005 | 0.891 | 2.0 | 84 | .047 | .21 |
| Step 3 (*R*^2^ = .099, *p* = .033) |  |  |  |  |  |  |  |
| Intercept | 1.65 | 1.46 | 1.83 | 17.8 | 83 | < .0001 |  |
| S-A Creativity test (originality) | 0.03 | -0.012 | 0.077 | 1.5 | 83 | .146 | .15 |
| CPS | 0.42 | -0.025 | 0.858 | 1.9 | 83 | .064 | .20 |
| S-A Creativity test (originality)  × CPS | 0.07 | -0.023 | 0.163 | 1.5 | 83 | .138 | .16 |

CI: confidential interval, LL: lower limits, UL: upper limits, CPS: creativity personality scale
