# Supplementary material for: Relationships among creativity indices: Creative potential, production, achievement, and beliefs about own creative personality
Source: PLoS One. 2022 Sep 28;17(9):e0273303. doi: 10.1371/journal.pone.0273303 (PMC9518913; doi:10.1371/journal.pone.0273303)
Supplement: Attachment — Submitted filename: Response to Reviewers_211022_English.docx [file pone.0273303.s006.docx]

I apologize for the delay in revising the manuscript. We thank that both reviewers provided us constructive comments and suggestions. Based on reviewers’ fruitful suggestions, we amended our manuscript thoroughly. We would appreciate if you find below our responses to each comments.

I found one mistake regarding an old manuscript. The old manuscript incorrectly handled zeros when converting creativity achievement values to logarithmic values. In the new manuscript, I did a logarithmic conversion using all values plus one. The results section has been amended accordingly. The results remain unchanged from the old manuscript.

**Reviewer #1:**

***Comment 1.*** *Unfortunately, this manuscript has flaws. It is superficial. The authors did not do their homework. For example, in section 2.3 the authors failed to connect their measures of creative potential with existing measures. They do mention Guilford, but not in a way that really helps. They could have admitted that one of their tasks is the Uses Test, another is the Consequences Test, and another is Product Improvement. Many people besides Guilford have used these and mentioning that would help readers understand if there is any generalizability.*

**Response 1.** The previous manuscript missed to explain the specific relationship between S-A creativity test and the previous divergent thinking tests. S-A creativity test was developed to assess DT under the supervision of Guilford and with reference to TTCT and the high reliability and validity were reported in previous research for Japanese participants. The subtests and the scoring process (in the four aspects: Fluency, Flexibility, Originality and Elaboration) are based on TTCT. The current manuscript illustrates this information with more precise reference to previous tests and research.

p. 8, l. 163: Creative potential was measured using the S-A Creativity test [42], standardized for Japanese participants. J.P. Guilford supervised the development of this instrument to assess DT based on the TTCT. It has high reliability and validity and can be administered to 4^th^ grade to university-level students [42]. S-A Creativity test scores have been applied to various research studies related to creativity in Japan. Specifically, the test showed the positive associations between personality and problem-solving in daily life [43]. It also showed the relationship between neurological and physiological data (e.g., [44,45]).

The test is composed of three types of subtests: Unusual Uses Task, Product Improvement, and Just Suppose in TTCT. The Unusual Uses Task subtest requires participants to generate unique ways of using typical objects, such as a newspaper, button, and chopsticks. For example, participants were asked the following question: “Other than reading, what other uses are there for newspapers? For instance, you may use them to wrap things.” The Product Improvement subtest requires participants to imagine the different ways a product, such as a TV, pot, or desk, can be improved. For instance, participants were asked, “What kind of TV can you imagine having? For example, a TV that can show 3D images. Write down as many as possible.” Finally, the Just Suppose subtest requires participants to imagine the consequences when something unimaginable happens, such as when there are no clocks in the world. For example, participants were asked, “What would happen if all the mice in the world disappeared? One possible consequence is that cats might become hungry.” Participants are given two minutes for a trial and five minutes for two questions in each subtest.

***Comment 2.*** *Even more problematic is that the current authors point to four indices, and some of these are not really a part of creativity theory. Rapidness and broadness, for example, are not theoretically tied to creativity. Rapidness sounds like something that is contrary to creativity. Gruber went into detail about how creative thinking takes time, and there are data from Mednick and other supporting that. Remote (original) associates take time and are not rapid. Beyond this, no operational definition of any of the indices is provided in this manuscript. This is a an enormous problem. Another problem is that a total score is formed from the four individual indices. That total score could be calculated in various ways, such as averaging across the four indices, or it could be an optimization of the four indices, but that is not described in the manuscript. There is debate in the research about totally vs using single indices, and usually the total score is rejected. All the authors do is refer to a external professional organization, one I have never heard of, even though I've been reading the creativity research for decades. You might say that there is much too much ambiguity here.*

**Response 2.** The previous manuscript inappropriately translated the four aspects (Fluency, Flexibility, Originality and Elaboration) into (Rapidness, Broadness, Originality and Elaboration). because we retranslated the relatively classic Japanese translation of the four aspects in 50 years ago to English without considering the correspondence to TTCT. Precisely, the S-A creativity test was developed with referencing the TTCT and the aspects should have been translated into Fluency, Flexibility, Originality and Elaboration. The present manuscript illustrated the correspondence of S-A creativity test and TTCT and appropriately translated the four aspects based on each definition and scoring procedure.

Although S-A creativity test generally use the sum of Originality and Elaboration score because of the higher correlation of Fluency with Flexibility and Elaboration, TTCT discouraged to use the total score of the aspects. Further, the reliabilities of each scoring should be clarified in each sample. Therefore, we asked the professional organization to show the raw scores of the test in the current sample and calculated the reliabilities and correlations. As a result, the reliabilities of Flexibility and Originality were less than .70, which may distort the estimation of true correlations. In addition, the correlation of Fluency and Elaboration was quite high (r = .71), which is difficult to discriminate the two aspects. Therefore, the current study decided to use the only Fluency for the further analyses.

p. 9, l. 170: The test is composed of three types of subtests: Unusual Uses Task, Product Improvement, and Just Suppose in TTCT. The Unusual Uses Task subtest requires participants to generate unique ways of using typical objects, such as a newspaper, button, and chopsticks. For example, participants were asked the following question: “Other than reading, what other uses are there for newspapers? For instance, you may use them to wrap things.” The Product Improvement subtest requires participants to imagine the different ways a product, such as a TV, pot, or desk, can be improved. For instance, participants were asked, “What kind of TV can you imagine having? For example, a TV that can show 3D images. Write down as many as possible.” Finally, the Just Suppose subtest requires participants to imagine the consequences when something unimaginable happens, such as when there are no clocks in the world. For example, participants were asked, “What would happen if all the mice in the world disappeared? One possible consequence is that cats might become hungry.” Participants are given two minutes for a trial and five minutes for two questions in each subtest.

The test is scored by trained judges on four aspects: Fluency, Flexibility, Originality, and Elaboration. Fluency is a measure of the ability to generate many ideas. It is evaluated by the number of responses, excluding those that are inappropriate or difficult to interpret. Flexibility is the ability to generate ideas from a wide range of perspectives. It is assessed by the number of categories in which ideas are generated based on the criterion table or equivalent judgment. The criterion table indicates a particular answer’s classification and how many points it receives. Originality is the ability to generate ideas different from those of others. It is evaluated based on the total number of categories weighted by the criterion table or an almost equivalent judgment; the criterion table indicates how many points should be given based on how rare the responses are in each category. Finally, Elaboration is the ability to think concretely about ideas. It is evaluated based on the total number of responses weighted by the criterion table or equivalent judgments. Torrance [46] initially established these four aspects as perspectives for evaluating ideas based on the elements of DT proposed by Guilford [9]. An external professional organization evaluated all of the ratings (Tokyo Shinri Corporations) [42].

p. 13, l. 258: The scores for Fluency and Flexibility highly correlated with those for Elaboration in the past surveys for Japanese. Therefore, the total creativity score was defined as the sum of the raw score of Originality and Elaboration in the S-A Creativity test. However, Guilford [9] viewed DT as multidimensional, and Torrance [46] discouraged researchers from using the total score of the four aspects. Since the use of the total score of the TTCT is controversial (e.g., [31,49]), the current study first checked the reliability of scoring of each aspect in the current sample, then examined the relationships among the aspects. Although the reliability score is required to be more than .80, or at least more than .70 in psychometric assessment [50], the current results showed that the reliability scores (Cronbach alpha) of some evaluation aspects were not acceptable (Fluency: .80; Flexibility: .67; Originality: .56; Elaboration: .77). On the other hand, the reliabilities of Fluency and Elaboration were adequate. Still, the correlation of the two aspects was relatively high (r(86) = .889, p < .0001). Therefore, the current study used the score for Fluency as the most reliable rating with similar characteristics to the other perspectives in subsequent analyses.

***Comment 3.*** *The authors would have been better off if they had a better sample of measures and tasks of creative potential. Right now they have three varied measures. It is much more common to use several items within any one type of test. It is more common for example, to have three Product improvement items, whereas the present authors only have one. Research often has three product improvement items and then three Uses items and then three Consequences items, or something like that. This allows authors to check the inter item reliability of their tests. Right now the authors have used only three items, and they are three items from three different kinds of tests, so you couldn't reasonably expect nor statistically check reliability. Again you could say that there is much too much ambiguity, here about reliability.
Ideally the authors would check reliability with their own sample because reliability always varies from sample to sample, and it is not satisfactory to simply report that they are using a measure that has demonstrated real reliability in the past with other samples. The Editor should watch for this in a revision. Do not allow them to simply refer to previous reliability.*

**Response 3.** The previous manuscript could not explain the specific procedure and scoring process in an appropriate way. The S-A Creativity test included three subtests that correspond to Unusual Uses Test, Product Improvement and Just Suppose. Participants are provided two minutes for a trial and five minutes for two questions in each subtest, which were scored in four aspects. As mentioned above, we also calculated the reliabilities of scoring in the four aspects. With considering the reliabilities of each scoring and the relationships between the four scores, the current study decided to use only Fluency for the subsequent analyses. These information were demonstrated in the procedure and results sections.

p. 9, l. 170: The test is composed of three types of subtests: Unusual Uses Task, Product Improvement, and Just Suppose in TTCT. The Unusual Uses Task subtest requires participants to generate unique ways of using typical objects, such as a newspaper, button, and chopsticks. For example, participants were asked the following question: “Other than reading, what other uses are there for newspapers? For instance, you may use them to wrap things.” The Product Improvement subtest requires participants to imagine the different ways a product, such as a TV, pot, or desk, can be improved. For instance, participants were asked, “What kind of TV can you imagine having? For example, a TV that can show 3D images. Write down as many as possible.” Finally, the Just Suppose subtest requires participants to imagine the consequences when something unimaginable happens, such as when there are no clocks in the world. For example, participants were asked, “What would happen if all the mice in the world disappeared? One possible consequence is that cats might become hungry.” Participants are given two minutes for a trial and five minutes for two questions in each subtest.

The test is scored by trained judges on four aspects: Fluency, Flexibility, Originality, and Elaboration. Fluency is a measure of the ability to generate many ideas. It is evaluated by the number of responses, excluding those that are inappropriate or difficult to interpret. Flexibility is the ability to generate ideas from a wide range of perspectives. It is assessed by the number of categories in which ideas are generated based on the criterion table or equivalent judgment. The criterion table indicates a particular answer’s classification and how many points it receives. Originality is the ability to generate ideas different from those of others. It is evaluated based on the total number of categories weighted by the criterion table or an almost equivalent judgment; the criterion table indicates how many points should be given based on how rare the responses are in each category. Finally, Elaboration is the ability to think concretely about ideas. It is evaluated based on the total number of responses weighted by the criterion table or equivalent judgments. Torrance [46] initially established these four aspects as perspectives for evaluating ideas based on the elements of DT proposed by Guilford [9]. An external professional organization evaluated all of the ratings (Tokyo Shinri Corporations) [42].

p. 13, l. 258: The scores for Fluency and Flexibility highly correlated with those for Elaboration in the past surveys for Japanese. Therefore, the total creativity score was defined as the sum of the raw score of Originality and Elaboration in the S-A Creativity test. However, Guilford [9] viewed DT as multidimensional, and Torrance [46] discouraged researchers from using the total score of the four aspects. Since the use of the total score of the TTCT is controversial (e.g., [31,49]), the current study first checked the reliability of scoring of each aspect in the current sample, then examined the relationships among the aspects. Although the reliability score is required to be more than .80, or at least more than .70 in psychometric assessment [50], the current results showed that the reliability scores (Cronbach alpha) of some evaluation aspects were not acceptable (Fluency: .80; Flexibility: .67; Originality: .56; Elaboration: .77). On the other hand, the reliabilities of Fluency and Elaboration were adequate. Still, the correlation of the two aspects was relatively high (r(86) = .889, p < .0001). Therefore, the current study used the score for Fluency as the most reliable rating with similar characteristics to the other perspectives in subsequent analyses.

***Comment 4.*** *Another concern is that the CAT method of judging products was used. And there are various problems discussed in the literature when using the cat. Various studies have shown that the results of CAT researech do not generalize: ratings from one group of judges are not well correlated with ratings from other groups of judges. Admittedly the CAT is often used but that doesn't mitigate the problem.*

**Response 4.** The previous manuscript could not specify the generalizability of the CAT results. The current study measured creative potential and production in a certain group with using the method of CAT. However, as you mentioned, the scoring results could be different if the other experts rated the product creativity. The present manuscript discussed this point as a limitation of generalizability of the current study in the discussion section.

p. 22, l. 433: The present study also examined creative production using the CAT method, criticized for its generalizability [35]. Plucker et al. [34] implied that the CAT includes a rating process by experts in the domain, and the validity of the rating process is still under consideration. As the current study failed to obtain an acceptable level of reliability for the Haiku rating, the CAT method for rating creative production cannot be generalized according to each area, rater type, culture, and society. Since Haiku is a unique Japanese literary expression, it is necessary to refine the product evaluation method in the future. Future studies should also examine the validity of the CAT method more specifically.

***Comment 5.*** *This research is not well tied to previous research. Indeed, the authors argue that the connection of potential to productivity has not really been studied, but as a matter of fact I know of several dozen studies which has explored that connection. It is a straw argument to say that it has not been studied. The present authors do cite a few studies, such as the longitudinal work using the Torrance data, but what of the very important seminal study of Wallach and Wing? And then Wallach did follow up studies, as did Nathan Kogan. Sure, these were done many years ago, but they arere quite meaningful and, again, it is incorrect to say that the connection has not been studied. results, period. I would also suggests that they look at the work of Roberta Milgram on potential as connected to actual creative productivity and performance, and Plucker’s re-analysis of the Torrance data, and Runco’s 2-3 studies of creative potential connected to creative behavior. It is poor scholarship to overlook so much relevant research.*

**Response 5.** The previous manuscript could not review the previous research on creativity. We appreciated your suggestion of previous articles and reviewed further research as long as we can access. Then, we reviewed the previous findings and reconsidered the findings of the current study. We believe that the current study has implications to illustrate the specific relationship of creative potential, production, achievements and personality. In addition, these relationships in a young adult group are suggestive to understand the interdisciplinary studies on creativity because such studies separately examine the relationships between a variety of creativity indices and biological measures in each study. The detailed review and the significance of the current study were specified in the introduction section.

p. 3, l. 54: Psychologists have measured such test scores to assess individuals’ creative potential, which is expected to relate to and predict their creative production and performance due to the Product factor of the 4 Ps theory [2]. However, this association between creative potential and performance is weakly positive in most studies conducted on children to adults (e.g., [13–17]). The predictive role of creative potential has also been examined but remains controversial. Kogan and Panvoke [13] showed that children’s creative potentials correlated with their accomplishments in the 5th but not in the 10th grade. The 18-year follow-up study also showed that the creative potential of high school students was only associated with specific areas of creative achievements [18]. Torrance [10] designed the Torrance Test of Creative Thinking (TTCT) to indicate creative potential that promotes creative behaviors. In addition, previous empirical research with a 40- or 50-year follow-up found that creative ideation scores predict future creative achievement [19–21]. Further, it is suggested in a meta-analysis [22] and follow-up study [19] that the TTCT has a better predictive association of creative achievements than IQ. However, it has been argued that the link between creative potential and behavior is weak (r score is less than .30) and sometimes insignificant [23]. An examination of what causes this weak correlation follows

.….

In recent years, the number of interdisciplinary studies on the neurological and genetic basis of creativity (e.g., [38–41]) has increased significantly. However, each one employed a different creativity index. Therefore, clarifying the relationship between creativity and the rest of the human biological substrate, and that among creativity indices, will help in explaining the biological basis of creativity.

***Comment 6.*** *Then there is the fact that the Creative Achievement Questionnaire has also been criticized in a convincing fashion. Results from this measure tend to be skewed, and the theoretical basis for the questions within the CAT have been criticized a number of times. Those questions were nominated by a small group of judges and are therefore limited and potentially biased. There is a much better measure called the Creative Activity and Accomplishment Checklist (CAAC), which was developed a long ago by Holland, and then refined in a dozen or more studies since then. Statistically and theoretically it is a much present better measure than the CAQ. An and then Paek have recently published using the newer, better CAAC. Why do I mention this? I know the present authors will not want to go back and collect new data with the better measure, but their choice and their ignoring the problems with the CAQ is another indication that they did not really do their homework. The authors look a bit foolish when they claim on line 47 “that most of the studies measuring creative behavior use the Creative Achievement questionnaire or the Biographical Inventory." The CAAC has been used 3-4 times as often as the CAQ, no doubt because it is a better measure. AND the BIC is really just a copy of the CAAC.*

**Response 6.** The previous manuscript did not mention the CAAC. As you mentioned, however, there are a variety of measurements of creative achievements other than CAQ. The current study chose CAQ because it was one of the popular measurements of creative achievement and because it was composed of questions that is applicable for Japanese participants. Still, the score of CAQ is often skewed (actually it was skewed in the current study) as you mentioned. The present manuscript warned this problem as the limitation of the current study and suggested that researchers should consider to select the other measurements or to consider the way to collect the appropriate participants in each research objective.

p. 19, l. 366: As in previous research that focused on creative potential and achievements (e.g., [13,14,16,17,19]), the current study only showed the positive association between creative potential and the total achievements score. This study used the CAQ, a popular measure of creative achievements. However, the distribution is often skewed depending on the nature of the target population and the data collection method. Since we collected study data from a relatively small group (fewer than 100 people), the overall scores could be used in the analysis. However, another measure for creative achievements should be considered when addressing the problem of not using the scores for each domain. As there are various measures to assess creative achievements in general and specific domains (e.g., Creative Activity and Accomplishment Checklist [24]; Runco Ideational Behavior Scale [53]; Biographical Inventory of Creative Behaviors [26]; Kaufman Domains of Creativity Scale [54]; Inventory of Creative Activities and Achievements [55]), future studies should translate such measures into various languages and conduct creativity research for diverse cultural background groups. Further, although it is often difficult to gather sufficient participants with higher creative achievements, a recent study proposed using a comparison group. Researchers could control variables of interest such as age, gender, intelligence, and creative potential in the higher and lower creative achievement groups [56]. Such a data collection method could be promising for future creativity research.

***Comment 7.*** *The authors claim that the link between creative potential and behavior is less than .3. That is often true, but there is research showing the connection using measures of creative potential, that are similar to those used here in with results of up to .55. Runco (1986) used mult regression with tests similar to the ones used herein and the criterion measure mentioned above and found that .55. Here again I don’t think the authors did a good job looking at previous research. One more example: Plucker did include a personality trait in his study of divergent thinking (creative potential) and creative behavior. He made the important point that tests of potential are often not aligned with the criteria used in the research. That would include the CAQ. Plucker et al. used a criterion that was aligned with the predictors–a criterion based on ideation (which is what the predictors measure).*

**Response 7.** The previous manuscript lacked enough explanation of the statistics. As you mentioned, Runco (1986) showed the moderate correlation with creative potential and achievement in a specific domain within the gifted children. The current study was conducted an experiment for young adults because the finding could be suggestive for understanding the recent interdisciplinary research on creativity. In the amendment manuscript, seminal multi-regression analyses for creativity indices with referencing the Plucker et al (2010) as you suggested.

p. 14, l. 280: Next, we examined the moderating effect of the CPS score on the association between creative potential and creative behavior. First, a multiple regression analysis was conducted with the creative production of art as the objective variable and creative potential score, CPQ score, and the interaction term between creative potential score and CPQ score as explanatory variables (Table 2). In Step 1, we examined the main effects of creative potential score on creative products and found that creative potential score had a positive effect (β = .26, t(84) = 2.4, p = .018). In Step 2, the CPS score was added to the explanatory variables, the effect of the fluency score remained (β = .24, t(83) = 2.3, p = .027) but there was no effect of the CPS (β = .16, t(83) = 1.5, p = .125). In Step 3, the interaction term between the creative potential score and the CPS score was added to the explanatory variables. Still, there was no interaction effect between the creative potential score and the CPS score (β = -.17, t(82) = -1.6, p = .117).

In addition, a multiple regression analysis was conducted with the CAQ score as the objective variable, creative potential and CPS scores as the explanatory variables, and the interaction term between the creative potential score and the CPS score (Table 3). In Step 1, we examined the main effects of the creative potential score on the CAQ score and found that the creative potential score had a positive effect (β = .31, t(85) = 3.0, p = .004). Next, the results of the analysis in Step 2, in which the CPS score were added to the explanatory variables, showed a positive effect of the creative potential score (β = .29, t(84) = 2.8, p = .006), but no in the CPS score (β = .20, t(84) = 2.0, p = .053). In Step 3, the interaction terms of the creative potential score and the CPS score were added to the explanatory variables, showed an interaction effect between the creative potential score and the CPS score (β = .33, t(83) = 3.5, p = .001, ΔR2 = .110). The simple main effect test results showed that the creative potential score had a positive effect on the CAQ score in the high CPS group (β = .68, t(83) = 4.6, p < .0001, Figure 5). However, the creative potential score had no effect in the high CPS group (β = -.04, t(83) = 0.3, p = .767).

***Comment 8.*** *I did not see probability level levels or degrees of freedom in the regression table. I am accustomed to APA tables, which require that all information is provided within each table; that the table is self-contained, self-sufficient. The authors report probabilities in the body of the paper but in the Tables. Degrees of freedom seem to be missing here and there (e.g., line 214).*

**Response 8.** The previous paper did not include information on degrees of freedom and probability. In this manuscript, we have added a table that covers information about these, and the results of the regression analysis are described in the text, including the degrees of freedom.

see Table 2 Table 3 and Results part:

***Comment 9.*** *I would not put divergent thinking in the Person category of the 4Ps. It is a process measure (how ideas are associated with one another) OR a measure of products (ideas = products). The Person category = traits and the like. DT is not a trait. It reflects a capacity to produce ideas, often original. This is a small point, I know, but if the ms is revised, I would change this. (And there is a 6P model, by the way, called the hierarchical model, but that too is a small point.)*

**Response 9.** After reviewing the contents of the 4P’s theory, as you pointed out, DTs are positioned to measure the cognitive process during idea generation. Therefore, in the revised paper, we discussed the relationship between creative potential as Process, production and achievements as Product, and personality as Person.

p. 20, l. 406: For instance, creativity can be distinguished depending on whether it is attributable to the Person, Process, Press (environment), and Product (4 P’s theory) [7]. This 4 P model has been further developed into the 6 P model [61], adding Purpose and Problems. The current study examined the associations of four creativity measures: creative potential, production, achievement, and personality, which correspond to Person (personality), Process (creative potential), and Product (production and achievements). The current findings revealed the associations between Process and Product and the moderating role of personality in this relationship. The reason creative production and achievements were not correlated in the current study could be due to the misalignment in comparison areas, such as production art and total score for achievement.

**Reviewer #2:**

***Comment 1.*** *The methodological approach could be better explained. For someone who doesn’t have knowledge about descriptive statistics and measurement variables, the graphics are difficult to understand.*

**Response 1.** The previous manuscript did not fully explain the variables measured and the statistical explanations. In the revised manuscript, I provided more detailed and clear explanation.

p. 11, l. 234: A series of self-report questionnaires measured participants’ creative achievement and creative personality. The first questionnaire was the CAQ by Carson et al. [25], which measured achievement in the 10 creative domains (fine arts, music, dance, architectural design, writing, comedy, invention, science, theater and film, and cooking). Total achievement scores in all domains were used in the analysis. The second was the CPS [37], which consists of 20 items selected from the International Personality Item Pool [47,48], which is a 5-point Likert scale (1: not at all to 5: very much) and measures creativity in general and in specific domains (e.g., science, interpersonal communication, writing, art). In this study, the overall mean was used for the analysis.

p. 12, l. 244: The descriptive statistics of the S-A Creativity test, creative production (in fine arts), total score of the CAQ, and the CPS score are shown in Table 1. The distribution of scores for each task is illustrated in Figure 3. A Shapiro-Wilk test was used to test whether scores on each measure of creativity were normally distributed, including fluency (W = 0.97, p = .053), flexibility (W = 0.98, p = .102) and originality (W = 0.98, p = .402), elaboration (W = 0.99, p = .813), creative production (W = 0.98, p = .180) and CPS score (W = 0.98, p = .196) were normally distributed, but the creative achievement score was not (W = 0.77, p < 0.0001). Therefore, log-transformed values of the creative achievement scores were used in the analysis.

***Comment 2.*** *It would also be nice to have some pictures of the “creative productions”: for example, a haiku poem or a cut out paper object.*

**Response 2.** In the previous manuscript, there were no images of paper cutouts or poems, which may have made it difficult to get a concrete image. In this paper, I have included images of paper cutouts as Figure 2 for each theme.

See Figure 2

***Comment 3.*** *The following formal structure would make the paper clearer:*

1. Introduction
1.1. Approaches to the Creativity Concept
1.2. Ways of Measuring Creativity Indices
1.3. Research Objectives \n

2. Methodological Approach \n
2.1. Participants
2.2. Procedure
2.3. Creative Potential Test
2.4. Creative Production Task
2.5. Rating Product Creativity

3. Results

4. Discussion
4.1. Impact of Personality on Creative Potential and Behaviour \n
4.2. Relationships between Creativity Indices \n
4.3. Conceptualisation of Creativity \n

5. Limitations and Future Studies

**Response 3.** The structure of the paper was not clear, so we followed the format you gave me and considered the structure. As you pointed out, it was necessary to describe the research approach to creativity in the introduction. Due to the flow of the paper, I could not add a section, but I added the contents related to the approach to the introduction.

p. 3, l. 40: Creativity is a uniquely human ability that psychological researchers have claimed is demonstrated by each individual in their everyday lives [1,2]. However, because it has a variety of attributes due to conceptual vagueness, researchers have classified the types of creativity according to various approaches ranging from cognitive and personality approaches to systems and sociocultural theories (e.g., [3–7]). For instance, the 4 Ps theory claims that creativity could be attributed to the Person (individuals’ cognitive and metacognitive traits), Process (psychological process to perform creative acts), Press (environment and situations that produce creativity), and Product (specific works and achievement to demonstrate creativity) [8]. While researchers have developed various instruments and methods to measure creativity based on such theoretical frameworks, the relationships among creativity indices remain unclear.

***Comment 4.*** *My last observation is related to the approach to Creativity. In this paper elements such as the social or cultural background of a creative individual in relation to ‘creative potential’ and ‘behaviour' are not considered. The authors of the study could include the systemic perspective of creativity which considers several factors, even including the role of the specialists who evaluate the creative achievements. I recommend reading articles or books from Csikszentmihalyi about the systemic perspective of Creativity.*

**Response 4.** As you pointed out, socio-cultural background is an important factor in creativity research. In fact, creative achievements are especially those that are produced by each research collaborator as they are influenced by the domain, society, and culture. This is a part of the press of the 4P's theory, but we could not examine this influence in this study. Therefore, in this paper, I have explained this issue as a limitation of the research, citing the Systems Model.

p. 21, l. 415: Although the current study focused on three of the 4 P models, Press is also essential in considering the relationships among creativity indices. It is evident that Person and Process change depending on the environment. Still, indices, such as creative production and creative achievement, measured in this study, include evaluations from others. This evaluation process is influenced by domain, culture, and society, as suggested by the systems model in Csikszentmihalyi [4]. The systems model suggested that creative activity and products were evaluated by experts in a specific domain and people belonging to a society and culture. The creative activities and products were situated within domains, a society, and culture through such processes. Future studies should illustrate such an influence by Press on Process and Product. For example, we could research the relationship among various creativity factors integrating creativity conceptualization.
