# Supplementary material for: Relationships among creativity indices: Creative potential, production, achievement, and beliefs about own creative personality
Source: PLoS One. 2022 Sep 28;17(9):e0273303. doi: 10.1371/journal.pone.0273303 (PMC9518913; doi:10.1371/journal.pone.0273303)
Supplement: Attachment — Submitted filename: Response to Reviewers_220412.docx [file pone.0273303.s007.docx]

We appreciate that the editor and both reviewers carefully checked our manuscript and provided us suggestive comments. According to the reviewers’ suggestions, we added more detailed descriptions in our manuscript. We would appreciate if you find below our responses to each comments.

**Reviewer #1**:

*The study has a good objective but the measures are not good (the CAT) and there are some questionable decisions (not examining all DT indices). I suppose researchers may learn something from this project, but it disappoints me that relevant research was not cited (e.g., Wallach and Wing) in the introduction and that the methodological issues mentioned earlier and above were not addressed.*

**Response**. As you pointed out in the first peer review, unreliable indicators could lead to regression dilution and reduction in testing power (Spearman, 1904). Since it is clearly statistically inappropriate to use unreliable indicators for regression analysis, Haiku CAT could not be used in the subsequent analysis. Similarly, flexibility and originality of DT could not be included in the regression analysis due to reliability issues. Further, we found that the fluency and flexibility were highly correlated. Therefore, they could not be included in the regression analysis simultaneously. However, as your suggestion, it is needed to report about all sub-indicators of TTCT (Torrance, 1979; Kim, 2008). Therefore, we have added the results of Elaboration to the text. Because of the low reliability coefficients for flexibility and originality, the results of the analysis are presented in the Supporting information without mentioning them in the main text.

p. 13, l. 260: Since the use of the total score of the TTCT is controversial and reporting of all four sub-scales is encouraged [31,49], the current study first assessed the reliability of scoring for each aspect in the current sample and then examined the relationships among these aspects. A reliability score of greater than .80—or .70 in psychometric assessment—is required to avoid regression dilution and reduction in testing power [50,51]. Thus, the current results showed that the reliability scores (Cronbach’s alpha) of fluency and elaboration were adequate, whereas those of other evaluation aspects were not (fluency: .80; flexibility: .67; originality: .56; elaboration: .77). Furthermore, the correlation between fluency and elaboration was relatively high (r(86) = .889, p < .0001). Therefore, as the subsequent analyses could not include DT sub-scales simultaneously, the scores for fluency and elaboration were used individually because these were the most reliable and such aspects demonstrated similar characteristics to the others. The results of the analysis of flexibility and originality, which are not mentioned in the text due to their low reliability, are included in the supporting information (Table S1, Table S2, Table S3, Table S4).

p. 17, l. 322: We found similar results using elaboration as creative potential. A multiple regression analysis was conducted using creative production of art as the objective variable and elaboration, CPS score, and the interaction term between elaboration and CPS score as explanatory variables (Table 4). In Step 1, we found that elaboration had a positive main effect on creative production (β = .31, t(84) = 3.0, p = .004). In Step 2, CPS score was included as an explanatory variable and the effect of elaboration remained (β = .30, t(83) = 2.9, p = .004), but CPS exhibited no effect (β = .18, t(83) = 1.8, p = .080). In Step 3, the interaction term between the creative potential (elaboration) and CPS score was included as an explanatory variable. However, there remained no interaction effect between elaboration and CPS score (β = -.11, t(82) = -1.1, p = .294).

p. 18, l. 336: Further, a multiple regression analysis was conducted with CAQ score as the objective variable and elaboration, CPS score, and the interaction term between the elaboration and CPS score as explanatory variables (Table 5). In Step 1, we found that elaboration had a positive main effect on CAQ score (β = .23, t(85) = 2.2, p = .031). The results of the analysis in Step 2, in which CPS score was included as an explanatory variable, showed a positive effect of elaboration (β = .23, t(84) = 2.2, p = .031) and CPS score (β = .23, t(84) = 2.2, p = .032). In Step 3, the interaction term of elaboration and CPS score was included as an explanatory variable, which showed an interaction effect between elaboration and the CPS score (β = .39, t(83) = 4.0, p < .0001). The simple main effect test results showed that elaboration had a positive effect on CAQ score in the high CPS group (β = .70, t(83) = 4.6, p < .0001, Fig 5B) wever, elaboration exhibited no effect in the low CPS group (β = -.09, t(83) = -0.7, p = .482).

p. 24, l. 466: Although it is standard to report on all sub-indicators of TTCT [31,49], originality and flexibility were not included in the current analyses due to inadequate reliability. Since these are two core concepts in DT, further research is necessary to examine whether these aspects produce similar results. The low reliabity of these sub-indicators was caused by issues with the Japanese TTCT scoring process.

We appreciate that your comments enabled us to learn a lot about creativity measurements. In fact, we have fully realized that the measurements in our study have a lot of limitations. Therefore, we demonstrated the limitations in the last amendments according to your comments. However, as you suggested, our description did not sufficiently address how researchers should measure creativity in the future study. Therefore, we have added a more detailed description about it as our maximum possible response in this manuscript. In addition, based on the limitations of this research, we are now starting a new project to review the issues in Japanese creativity research and introduce appropriate scoring flows for creative potential (e.g., Reiter-Palmon et al., 2019) and to develop a scoring system with applying natural language processing. We will build the basis of creativity research with appropriate procedures.

p. 24, l. 466: Although it is standard to report on all sub-indicators of TTCT [31,49], originality and flexibility were not included in the current analyses due to inadequate reliability. Since these are two core concepts in DT, further research is necessary to examine whether these aspects produce similar results. The low reliability of these sub-indicators was caused by issues with the Japanese TTCT scoring process. The study scales were translated and redeveloped from the TTCT for Japan. However, we outsourced scoring to an external organization. Thus, the scoring process was closed. Tests like the TTCT, translated worldwide, are convenient and easy to use. Still, researchers should pay attention to the validity and reliability of the scoring process in each test. Future studies in Japan should utilize a new scoring system—as described below—to ensure reliable and valid scoring.

p. 25, l. 484: Recently, researchers have indicated a procedural issue regarding creativity measurement in which scorers evaluate ideas or products. In scoring DT tasks or the CAT, the scorer should be trained in DT scoring [63] or have sufficient knowledge and experience in a particular creative domain [3]. Studies have shown that despite recruiting such scorers, their scoring generally became burdensome, especially with a large sample [64,65]. To solve these issues, new scoring systems using Latent Semantic Analysis (LSA) [66] have been proposed in recent creativity research [67,68]. LSA scoring has the potential to enable researchers to automatically score DT or literal products with high reliability and validity [64]. As such scoring systems have not yet been proposed in Japanese, future studies are necessary to develop these systems and conduct creativity research based on reliable and valid Japanese measurements.

Although we cited the Wallach & Wing(1969) in the introduction in the previous revision, the description may not have been able to adequately account for their contributions. In this paper, we have added a specific description of the Wallach & Wing (1969) study in the introduction.

p. 3, l. 49: However, this association between creative potential and performance is weakly positive in most studies conducted on children to adults [13–17]. The predictive role of creative potential has also been examined but remains controversial. Wallach and Wing [15] showed a positive association between creative potential and creative performance. They conducted an experimental survey of 503 freshman university students and found that creative potential predicted non-academic outcomes that IQ and standardized tests alone did not. Subsequent longer-term longitudinal studies have examined the predictive power of DT in more detail. Kogan and Panvoke [13] showed that children’s creative potentials correlated with their accomplishments in the 5th but not in the 10th grade. The 18-year follow-up study also showed that the creative potential of high school students was only associated with specific areas of creative achievements [18]. Torrance [10] designed the Torrance Test of Creative Thinking (TTCT) to indicate creative potential that promotes creative behaviors. In addition, previous empirical research with a 40- or 50-year follow-up found that creative ideation scores predict future creative achievement [19-21]. Further, it is suggested in a meta-analysis [22] and follow-up study [19] that the TTCT has a better predictive association of creative achievements than IQ. However, it has been argued that the link between creative potential and behavior is weak (r score is less than .30) and sometimes insignificant [23]. An examination of what causes this weak correlation follows.
