# Supplementary material for: Relationships among creativity indices: Creative potential, production, achievement, and beliefs about own creative personality
Source: PLoS One. 2022 Sep 28;17(9):e0273303. doi: 10.1371/journal.pone.0273303 (PMC9518913; doi:10.1371/journal.pone.0273303)
Supplement: Attachment — Submitted filename: Response to Reviewers_220801t.docx [file pone.0273303.s008.docx]

**Response to Reviewers’ comments**

We appreciate that the editor and both reviewers carefully checked our manuscript and provided us with suggestive comments. According to the reviewers’ suggestions, we added more detailed descriptions to our manuscript. We would appreciate it if you find below our responses to each comment.

**Reviewer #3**:

***Comment 1.*** *The justification for personality as a moderating variable is not clear to me. One issue I have is the use of the word “intervene” which to me suggests mediation and not moderation. Further, the description of the evidence does not necessarily suggest that personality is a moderator. This part of your introduction needs to be strengthened. I would also suggest that you focus specifically on the measure of personality or personality aspects you are including in your study. Personality can include multiple variables, and there is no reason to believe that findings from one personality characteristic would apply to another.*

**Response**. The last manuscript was vague in the justification for the role of the creative personality. As your suggestion, we avoided using the “intervene” and similar expressions. Further, we added the description that the current study focuses on CPS as individuals’ beliefs on creative personality and how the beliefs on creative personality affect the association between DT and creative production/achievements. Focusing on the role of beliefs in creative personality, we assumed that the beliefs could have mediating or moderating role between DT and creative production/achievements according to CBAA theory and its empirical findings. As creative personality can be considered as creative potential (Lubart et al., 2013), it can be assumed that the creative personality beliefs cannot be the antecedents of DT, which is assumed as one of creative potential, too. Thus, we assumed the moderating role of creative personality beliefs on the relationship between DT and creative production/achievements.

As you mention, personality can have multiple variables. Although the current study applied CPS to capture the beliefs of various creative personalities, the findings cannot be applied to the other personalities. Thus, the discussion about the role of personality was amended in the current manuscript.

**P. 6, l. 109:** Here, a question is begged: what can explain these findings on the inconsistent and weak association between DT and creative production and achievements? A recent theory by Karwowski and Beghetto [38] provides an outlook on this matter, proposing that individuals actualize their creative potential based on their personal intentions. They named the theory Creative Behavior as an Agentic Action (CBAA), positing that creative self-beliefs (which encompasses creative self-efficacy and perceived value of creativity) is a personal factor that mediates (through creative self-efficacy) and moderates (through perceived value of creativity) the relationship between DT and creative behavior [38, 39]. Hence, CBAA proposes that individual traits and attitudes toward creativity can influence the relationship between DT and creative behavior. Importantly, these propositions of CBAA raise the question as to whether other characteristics at the individual level can influence this relationship.

Kaufman [40] suggested that self-assessed measures of creative personality (e.g., openness to experience and beliefs about own creative personality) can be used to perceive how individuals view their creativity in addition to measures of creative self-beliefs (e.g., creative self-efficacy and perceived value of creativity). That is, beliefs about own creative personality can have a mediating or moderating role on the association of DT with creative production and achievement. Since these trait-related measures of creativity are assumed to be conative dimensions of creative potential [14], they cannot be preceded by DT, namely, they cannot have a meditating role on the relation between DT and creative behavior. Here, an interrogation arises: can beliefs about own creative personality strengthen individuals’ actualization of DT into creative behaviors? The confirmation of such an assumption may provide support to the triangle relationship of the Person, Process, and Product dimensions.

**P. 22, l. 412:** According to the assumption by Lubart et al. [14], creative potential includes cognitive resources (e.g., DT) and conative resources (e.g., openness to experiences), and used DT and creative personality (openness) as psychological components. Then, these cited authors constructed a latent factor of creative potential by combining DT and openness, showing their impacts on creative production and achievements. This type of functional model of creative personality comprising DT and production and achievements is important when focusing on personalities, as personalities are considered to have close relationships with genetic and other innate factors (e.g., openness to experiences and novelty seeking) [59,60]. Meanwhile, when measuring personality traits using self-assessed scales, the personality scores show individuals’ own beliefs about each item. If we consider that the CPS measures beliefs about own creative personality, another function of conative resources of creative potential comes to light: Beliefs about own conative resources influence how DT is realized in actual creative behavior. Future research should examine the dual function of creative personality creative personality and beliefs about own creative personality in creativity actualization, as this may yield relevant data for better understanding the specific function of the Person dimension in the 4 Ps theory.

***Comment 2.*** *Justification for the selection of CPS as a personality variable is also limited. Just because the research has not been conducted, does not mean that it should. You should justify why that is a gap in our understanding of the link between personality and creativity.*

**Response.** The last manuscript did not fully explain why CPS should be applied to the current investigation. The reason why CPS was used in the current study is that the current study aimed to focus on the beliefs of a wide range of creative personalities. Since CPS was developed from the items related to creativity but not from the previous personality research, thus it covers a wider range of creative personality traits. This justification was described in the introduction section.

**P. 7, l. 139:** We used the self-assessed Creative Personality Scale (CPS) [41, 42] to examine beliefs about own creative personality because it comprises items that relate to various creative traits of thinking, problem-solving, and imagination. Although the CPS does not specifically assess personality, it does yield self-assessed data on a wide range of creative traits, allowing us to understand the moderating role of beliefs about own creative personality on the dimensions of creativity.

***Comment 3****. Further, the CPS as a measure of personality includes multiple personality traits, and as such is more difficult to interpret. This makes supporting its use and interpretation of the results more difficult. Using specific and more targeted personality variables (like the big 5, or specific traits such as tolerance for ambiguity or creative self-efficacy) would provide a better understanding of the relationship between personality and creativity.*

**Response.** The last manuscript did not explain the reason for using CPS other than openness or other creative personality. The current manuscript demonstrated that the current study applied CPS as the belief of creative personality because it was developed from personality items that relate to creativity. Of course, even if we applied CPS as beliefs of creative personality, the function of other personalities should be examined. Especially, personalities such as openness to experience and novelty seeking are important ones because they are closely related to genetic or innate factors. Thus, we described that the functions of such personality traits and beliefs of specific creative personalities should be further investigated in the future study in the Discussion section.

**P. 22, l. 412:** According to the assumption by Lubart et al. [14], creative potential includes cognitive resources (e.g., DT) and conative resources (e.g., openness to experiences), and used DT and creative personality (openness) as psychological components. Then, these cited authors constructed a latent factor of creative potential by combining DT and openness, showing their impacts on creative production and achievements. This type of functional model of creative personality comprising DT and production and achievements is important when focusing on personalities, as personalities are considered to have close relationships with genetic and other innate factors (e.g., openness to experiences and novelty seeking) [59,60]. Meanwhile, when measuring personality traits using self-assessed scales, the personality scores show individuals’ own beliefs about each item. If we consider that the CPS measures beliefs about own creative personality, another function of conative resources of creative potential comes to light: Beliefs about own conative resources influence how DT is realized in actual creative behavior. Future research should examine the dual function of creative personality creative personality and beliefs about own creative personality in creativity actualization, as this may yield relevant data for better understanding the specific function of the Person dimension in the 4 Ps theory.

***Comment 4****. In part you reason that you choose two different production tasks – one in visual art and one in writing. I will note a few concerns. First, due to low reliability, you had to omit one of the tasks. Second, both tasks are in the art domain. I would expect a much different relationship may emerge if you used very different domains such as everyday problem solving and art. One solution to both of these issues is to avoid the issue of multiple domains in the introduction, and note the concern of domain specificity in the discussion.*

**Response.** The last manuscript did not provide a valid solution to clarify whether or not there is a difference in the associations between the beliefs on creative personality, DT, and production/achievements across specific creative domains. Thus, as you suggested, the current manuscript avoided to emphasize the domain specificity problems in the introduction section. Otherwise, we discussed the necessity to examine the domain-specificity problem in the discussion section.

**P. 23, l. 433:** Furthermore, there are other creative domains (e.g., science, social activities, etc.) which have yet to be examined by scholars. Thus, future studies should explore the differences in the association between DT and creative production across different creative domains. Interestingly, the moderating role of beliefs about own creative personality was not consistent between creative behaviors (i.e., production and achievement). Instead, beliefs about own creative personality did not explain the increase in variance in creative production, nor was it significantly associated with creative production in fine arts. This low explanation from beliefs about own creative personality may be due to the settings of the creative production tasks proposed in the current study, which only dealt with an art domain. Accordingly, although the participants in this study had higher creative personality, they may not have been willing to utilize their DT in a cutouts task. In other words, beliefs about own creative personality regarding specific creative domains are likely to influence DT differently across tasks in different creative domains. Kaufman and Baer [42] showed a significant relationship between CPS and self-assessed creativity in specific creative domains, albeit the relationships were weak (e.g., r = .23 in the art domain). Thus, future research should focus on beliefs about own creative personality in specific creative domains when making domain-specific examinations of the impact of such beliefs on the relationship between DT and creative production.

***Comment 5****. The last section in the introduction about neuroscience seems out of place and not really relevant to the study. Clarity regarding measurement is critical for the field as a whole.*

**Response.** As your suggestion, the current study itself was not related to neuroscience. Thus, the current manuscript excluded the neuroscience parts.

***Comment 6****. Your sample size is too small. Research indicates that Correlational (and regression by extension) research does not provide stable results until 150-250 participants (see Schönbrodt & Perugini, 2013).*

*Schönbrodt & Perugini (2013). At what sample size do correlations stabilize?, Journal of Research in Personality, 47, 609-612.* [*https://doi.org/10.1016/j.jrp.2013.05.00*](https://doi.org/10.1016/j.jrp.2013.05.00)

**Response.** As you noted, the number of participants in this study (n = 88) may not be large. However, when we performed a post-hoc power analysis using G*Power 3.1, all of our main results exceeded a power of 0.8. These results indicate that 88 participants are not a small number. We have added the results of the sensitivity power analysis to the main text.

This study involved creative production tasks in two areas that were time and labor intensive for the participants. In addition, given the effort required to evaluate the experts, it was not possible to increase the number of participants in this study. However, as you noted, larger sample size is necessary for the stability of the correlation scores. Therefore, we have added this issue as a limitation of this study.

**P. 13, l. 273:** This study did not use a prior sample design. Therefore, we conducted a sensitivity power analysis using G*Power 3.1 [53]. For a correlation analysis between two variables with power set at 0.8, we could theoretically detect an effect size (ρ) greater than 0.289 with 88 participants, and greater than 0.291 with 87 participants.

Regarding moderation effect analyses, with power set at 0.8, we could theoretically detect an effect size (f2) greater than 0.091 with 88 participants, and an effect size (f2) greater than 0.092 with 87 participants.

**P. 28, l. 547:** Finally, although we tried to recruit the largest sample possible, we were not able to recruit more than 100 participants due to budget constraints and the potential burdens on participants and creative production task raters. Accordingly, although the current sample size (N = 88) has sufficient statistical power, it did not show sufficient stability for the estimates according to research by Schönbrodt and Perugini [73]; these authors posit that correlational studies should collect data from more than 150 participants for ensuring estimate stability. Thus, future researchers should try and replicate the current research with larger samples to confirm our findings.

***Comment 7****. You discuss domain issues, but even if you could test on both production tasks, the two are both art based(writing a haiku and cut out). In that respect, they are focused on two sub-domains within the art domain and the argument throughout for the importance of domains seems misaligned.*

**Response.** As you suggest, the last manuscript did not provide a valid solution to the domain specificity problem because the current study conducted creative production tasks only in two art domains. Thus, as you suggested, the current manuscript avoided focusing on the domain specificity problems in the introduction section. Otherwise, we discussed the necessity to examine whether or not there is a difference in the associations of beliefs on creative personality, DT, and production/achievements across different creative domains such as engineering, science, or leadership other than arts.

**P. 23, l. 433:** Furthermore, there are other creative domains (e.g., science, social activities, etc.) which have yet to be examined by scholars. Thus, future studies should explore the differences in the association between DT and creative production across different creative domains. Interestingly, the moderating role of beliefs about own creative personality was not consistent between creative behaviors (i.e., production and achievement). Instead, beliefs about own creative personality did not explain the increase in variance in creative production, nor was it significantly associated with creative production in fine arts. This low explanation from beliefs about own creative personality may be due to the settings of the creative production tasks proposed in the current study, which only dealt with an art domain. Accordingly, although the participants in this study had higher creative personality, they may not have been willing to utilize their DT in a cutouts task. In other words, beliefs about own creative personality regarding specific creative domains are likely to influence DT differently across tasks in different creative domains. Kaufman and Baer [42] showed a significant relationship between CPS and self-assessed creativity in specific creative domains, albeit the relationships were weak (e.g., r = .23 in the art domain). Thus, future research should focus on beliefs about own creative personality in specific creative domains when making domain-specific examinations of the impact of such beliefs on the relationship between DT and creative production.

***Comment 8****. There are a number of issues with your discussion of scoring of the different tasks.*

a. *Please provide additional clarity on the ratings of the divergent thinking tasks. You mentioned that they were scored by expert judges, and later by a professional organization. Is this the same or different? Do you have information on inter-rater reliability for this?*

**Response.** The last manuscript was unclear about DT scoring process. The DT rating was performed by one judge from a professional organization. The organization does not open the specific procedure of the scoring. However, according to the communication with the organization, in the scoring, one expert judge rated the participants’ responses in accord with the scoring chart created from sample data of the Japanese population. Thus, the organization did not provide inter-rater reliability information. This information and further specification of the procedure were added in the method section.

**P. 10, l. 199:** The test is scored based on four aspects: fluency, flexibility, originality, and elaboration. Fluency is a measure of the ability to generate many ideas, being evaluated by the number of responses excluding those that are inappropriate or difficult to interpret. Flexibility is the ability to generate ideas from a wide range of perspectives, being assessed by the number of categories in the ideas generated and according to a criterion table (i.e., indicating a particular answer’s classification and how many points it receives) or equivalent judgment [43]. Originality is the ability to generate ideas different from those of others, which is again evaluated based on a criterion table (i.e., indicating the frequency of occurrence of categories for each response) [43]. Those with a frequency of occurrence of less than 1% scored 2 points, of 1–5% scored 1 point, and of more than 1% scored 0 points [43]. Finally, elaboration is the ability to think concretely about ideas, being evaluated based on the total number of responses weighted by a criterion table (i.e., describing how well the response depicts the means and structure of the purpose or functions) or equivalent judgments [43]. Torrance [47] initially established these four dimensions for evaluating ideas based on the elements of DT as proposed by Guilford [13].

One trained judge from an external professional organization performed all of the ratings; the expert judge evaluated the participants’ responses while following the scoring charts developed according to data from a sample of the Japanese population (Tokyo Shinri Corporations) [43].

b. *Inter-rater reliability should be evaluated using Interclass Correlations (ICC, Shrout and Fleiss, 1979). Alphas are akin to ICC (3) and tend to be a more inflated measure of inter-rater reliability. The number of raters (5) also influences this. Therefore, an inter-rater agreement of .72 for the CAT is on the low end.*

**Response.** As you suggest, the ICC is a more appropriate measure to assess the raters’ agreement. Thus, we re-calculated intraclass-correlations, following the Cseh et al. (2019), Koo & Li (2016), and Shrout & Fleiss(1979). The current study assumed that each participant’s product was rated by a different 5 raters from a larger sample and the raters’ scores will be calculated into average scores. Therefore, the ICC (2, k) (Shrout & Fleiss, 1979; Koo & Li, 2016) was calculated using “irr” package for R (Gamer et al., 2019) based on a mean-rating (k = 5), absolute-agreement, 2-way mixed-effects model, which was an acceptable level of agreement. This procedure was applied to calculate inter-rater agreements in both of Haiku and cutout pieces evaluation. As the same as the last analyses, Haiku evaluation agreements were quite poor. In regard to the cutout pieces, as you noted, ICC (3, k) (which is the same as alpha) was high (.80) and ICC(2,k), which assumes absolute agreement, was smaller (0.75). However, we still observed a moderately high rate of rater agreement.

**P. 12, l. 243:** Following Amabile’s [20] procedure, factor analyses were performed for rating three Haikus of each participant by five raters. The eigen values (4.15, 1.08, 0.19, -0.02) suggested that the dimensions consisted of two factors and the reliabilities as a consistency were good (αs> .80). Further, the CAT scoring procedure requires inter-rater agreement [48]; although the current study calculated ICC(2, k) using the “irr” package for R [49] based on a mean-rating (k = 5), absolute-agreement, and 2-way mixed-effects model, the inter-rater agreements were quite poor (ICCs(2,k) < .20). Thus, the results of the Haiku ratings were not included for further analysis. (… ) Factor analyses were performed for rating two products by each participant by five raters. The eigen values (5.72, 0.46, 0.21, 0.08) suggested that the dimensions consisted of one factor and the reliability as a consistency was good (α= .95). Further, the CAT scoring procedure requires the inter-rater agreement [48]; the current study calculated ICC(2, k) into 0.75 [CI: 0.62-0.84] based on a mean-rating (k = 5), absolute-agreement, and 2-way mixed-effects model, which was an acceptable level of agreement.

c. *I found the description of the CAT ratings confusing. Were ratings given on each dimension? If not, what were the 10 dimensions used for? How were the 10 dimensions used to create a final score? Was reliability calculated across or within each dimension? Please provide more detailed information about the rating and scoring.*

**Response.** The procedure of CAT rating was unclear in the last manuscript. The expert judges rated each product using 10 dimensions respectively. According to Amabile (1983)’s CAT procedure, factor analyses were performed on the scores of two participants’ works rated by five raters on 10 dimensions. The results indicated the one factor solution; therefore, the subsequent analyses used the average score of 10 dimensions as the artistic production score. These procedures were described more specifically in the results section.

**P. 13, l. 256:** Factor analyses were performed for rating two products by each participant by five raters. The eigen values (5.72, 0.46, 0.21, 0.08) suggested that the dimensions consisted of one factor and the reliability as a consistency was good (α= .95). Further, the CAT scoring procedure requires the inter-rater agreement [48]; the current study calculated ICC(2, k) into 0.75 [CI: 0.62-0.84] based on a mean-rating (k = 5), absolute-agreement, and 2-way mixed-effects model, which was an acceptable level of agreement.

***Comment 9****. You state that you used the total CAQ score. There is a debate about whether the total should be used at all (see Silvia et al., 2012). Using the total score would require a justification which you currently do not provide. You could alternatively use the factor solution suggested by Carson and he colleagues to create 2 larger scales.*

**Response.** As your suggestion, we could have analyzed the factor structure of CAQ score; however, the sample size of the current study is only 88, which is below the sample size required in the factor analyses (e.g., Kline, 1993; COSMIN checklist). Therefore, the current study could not implement the factor analysis.

***Comment 10****. Your discussion about the total creativity score and summing was confusing. Since you did not do that, I would remove the mention of the sum, and just focus on the fact that you did not sum the scores because it is not appropriate to do so.*

**Response.** This discussion was added according to the previous discussion with the reviewers. In fact, the S-A creativity test organization provided the total score. Further, in the current review process, the other reviewer also suggested using the total score of DT. However, the previous studies discouraged to use of the total score of DT, we decided to describe our policy to analyze DT scores in the result section.

***Comment 11****. Some aspects of your discussion section are better suited for the introduction. For example, the discussion about conceptualizations of creativity fits better in the introduction. Specifically, a more detailed discussion the 4Ps and how they relate to the current study should be included in the introduction. The discussion should reiterate this but in a brief sentence.*

**Response.** The last manuscript did not fully demonstrate the typology of creativity. According to your suggestion and the other reviewer’s comments, the current manuscript added a more detailed discussion about 4Ps in the introduction and make the discussion section brief.

***Comment 12****. Language considerations – you seem to use CAT and production task interchangeably, the same applies to DT/TCTT/creative potential. The first is confusing because CAT is the rating process not the task. The second is confusing because creative potential is the theoretical concept and DT or TCTT are the tasks used to measure it. Please be consistent in their use in the appropriate place.*

**Response.** As you suggest, the last manuscript had the confusion in wording of concepts related to creativity (e.,g DT, creative production/achievement) and creative measures (e.g., S-A creativity test, creative production score, CAQ score). Thus, the current manuscript amended these confusing words thoroughly.

***Comment 13****.* There is a typo in author names for citation #37.

**Response.** We amended the typo.

**Reviewer #4**:

***Comment 1****. At a theoretical level, I think the paper suffer several weaknesses (see detail below).I think that it is problematic to call “creative potential” something that, in fact, boils down to divergent thinking. Creative potential is a broader concept than divergent thinking, see e.g. Lubart et al. 2013* [*https://files.eric.ed.gov/fulltext/EJ1301375.pdf*](https://files.eric.ed.gov/fulltext/EJ1301375.pdf)

**Response.** We appreciate that you provide the references. As you suggest, Lubart et al. (2013) suggested that creative potential includes various components such as divergent thinking or creative personality. Therefore, we amended the way to use the term “creative potential” in the current manuscript thoroughly. Further, this theoretical classification was demonstrated in the introduction discussion about 4Ps.

**P. 3, l. 54:** Creative potential, in turn, is expected to relate to and predict creative production and achievements, which relate to the Product dimension [16]. Following these theoretical assumptions, various researchers have examined the impact of creative personality (Person dimension) and creative thinking (Process dimension) on creative production and achievements (Product dimension). However, the relationships among these creativity indices remain unclear.

(…)

Since these trait-related measures of creativity are assumed to be conative dimensions of creative potential [14], they cannot be preceded by DT, namely, they cannot have a meditating role on the relation between DT and creative behavior.

**P. 22, l. 412:** According to the assumption by Lubart et al. [14], creative potential includes cognitive resources (e.g., DT) and conative resources (e.g., openness to experiences), and used DT and creative personality (openness) as psychological components. Then, these cited authors constructed a latent factor of creative potential by combining DT and openness, showing their impacts on creative production and achievements.

***Comment 2****. There have also been recent theoretical and empirical proposals concerning the articulation between personality, process and creative achievement, also in connection with the notion of creative potential, see*

*e.g. Fürst & Grin 2018* [*https://www.sciencedirect.com/science/article/abs/pii/S1871187117303334*](https://www.sciencedirect.com/science/article/abs/pii/S1871187117303334)

**Response.** We appreciate your providing further reference. This finding also supported us to understand the relationships between creative personality, DT, and production/achievements. We added a further discussion of the relationships by referring to this article in the discussion section.

**P. 22, l. 411:** For instance, Fürst and Grin used the score of openness/intellect as an index of creative personality [58]. According to the assumption by Lubart et al. [14], creative potential includes cognitive resources (e.g., DT) and conative resources (e.g., openness to experiences), and used DT and creative personality (openness) as psychological components. Then, these cited authors constructed a latent factor of creative potential by combining DT and openness, showing their impacts on creative production and achievements. This type of functional model of creative personality comprising DT and production and achievements is important when focusing on personalities, as personalities are considered to have close relationships with genetic and other innate factors (e.g., openness to experiences and novelty seeking) [59,60].

***Comment 3****. Major works in creative personality were also done by Hans Eysenck, see e.g. the book Genius: The Natural History of Creativity or*

*Eysenck 1993* [*https://www.tandfonline.com/doi/abs/10.1207/s15327965pli0403_1*](https://www.tandfonline.com/doi/abs/10.1207/s15327965pli0403_1)

**Response.** We appreciate your providing further reference. This book was informative to understand the characteristics of personality in creative genius. As a result of amending the manuscript, the current study described our focus on creative personality as the beliefs on creative personality.

***Comment 4****. When speaking of creative personality, it also seems essential to talk about the personality factor Openness, which is the main factor associated to personality. This seems especially crucial because the CPS is arguably a measure of Openness. (Most items of this scale are very similar to the key items of Openness, e.g. “Love to daydream”, “Like to solve complex problems”, “Have a vivid imagination”, etc.).*

**Response.** As you pointed out, the last manuscript did not describe the representative of creative personality such as openness to experience, tolerance of ambiguity, and so on. Thus, the current manuscript discriminated the difference between CPS and the other creative personality. This study focused on the CPS because it shows individuals’ beliefs on creative personality. Although CPS is, as you pointed out, quite a similar concept to openness, it covers the personality items that relate to creativity. Thus, it is useful to examine the role of beliefs of creative personality on the relationships between DT and creative production/achievements with applying CPS. However, this is the first step of examining the beliefs on creative personality role on the association between DT and creative production/achievements. Thus, the current manuscript demonstrated the necessity to further investigating the function of specific creative personality (i.e., openness to experience, tolerance of ambiguity and so on) in the discussion section.

**P. 22, l. 412:** According to the assumption by Lubart et al. [14], creative potential includes cognitive resources (e.g., DT) and conative resources (e.g., openness to experiences), and used DT and creative personality (openness) as psychological components. Then, these cited authors constructed a latent factor of creative potential by combining DT and openness, showing their impacts on creative production and achievements. This type of functional model of creative personality comprising DT and production and achievements is important when focusing on personalities, as personalities are considered to have close relationships with genetic and other innate factors (e.g., openness to experiences and novelty seeking) [59,60]. Meanwhile, when measuring personality traits using self-assessed scales, the personality scores show individuals’ own beliefs about each item. If we consider that the CPS measures beliefs about own creative personality, another function of conative resources of creative potential comes to light: Beliefs about own conative resources influence how DT is realized in actual creative behavior. Future research should examine the dual function of creative personality creative personality and beliefs about own creative personality in creativity actualization, as this may yield relevant data for better understanding the specific function of the Person dimension in the 4 Ps theory.

***Comment 5****. At the empirical level, the analysis reported in Table 2-3 and Table 4-5 seem redundant. It is the same results, once with Fluency and once with Elaboration. I would have simply used a total divergent thinking score combining fluency, flexibility, originality and elaboration. I understand that the authors were reluctant to do this. But still, I wonder what is the correlation between fluency and elaboration? And is this really necessary to perform two analysis that basically return the same results*

**Response.** In regard to the sum score, the previous study discouraged producing the total score of DT as described in the manuscript. Further, in the previous review process, the other reviewer also discouraged using the total score of DT. Thus, we decided to conduct the current analysis for each DT subscale and describe our policy to analyze DT scores in the result section. Thus, the Tables remained in the same manner.

***Comment 6.*** *In line with previous comment, I think that it would be a good thing to provide a correlation matrix of all variables in the study. Some of them are reported in the text, but it is not exhaustive and it is not convenient.*

**Response.** As your suggestion, we added a correlation matrix in Table 2 in the current manuscript.

***Comment 7.*** *P. 22, the authors mention the Runco Ideationnal Behavior Scale as a measure of creative achievement. This scale is not a measure of creative achievement, it is a measure of ideational “habits”, no item of this scale is focused on achievement whatsoever.*

**Response.** We appreciate your comment. We excluded the description of Runco Ideational Behavior Scale as a measure of creative achievement in the current manuscript.

***Comment 8.*** *At the very end of the paper, the authors say that future research should clarify the influence of personality on creativity. The literature on this topic is so vast and so much work have been done in this direction that such a comment is simply not acceptable as a way to close the paper!*

**Response.** As you suggested, the end of the last manuscript was not an appropriate discussion about personality and creativity. Thus, the current manuscript excluded the part.
